# Intensive Care Based Interventions to Reduce Family Member Stress Disorders: A Systematic Review of the Literature

**DOI:** 10.2478/jccm-2022-0014

**Published:** 2022-08-12

**Authors:** Sarah Love Rhoads, Thomas A. Trikalinos, Mitchell M. Levy, Timothy Amass

**Affiliations:** 1 Department of Medicine, Division of Pulmonary Sciences and Critical Care, University of Colorado, Denver, CO, USA; 2 Departments of Health Services, Policy, and Practice and of Biostatistics, and Center for Evidence Synthesis in Health, Brown University School of Public Health, Providence, RI, USA; 3 Department of Medicine, Division of Pulmonary, Critical Care and Sleep, Brown University, Providence, RI, USA; 4 Department of Veterans Affairs, Eastern Colorado Health Care System, Denver, CO; CU Anschutz: University of Colorado - Anschutz Medical Campus, Denver USA

**Keywords:** stress disorder, PICS-F, intensive care, family care

## Abstract

**Background:**

Increasing awareness of the emotional impact of an Intensive Care Unit (ICU) hospitalization on patients and their families has led to a rise in studies seeking to mitigate Post Intensive Care Syndrome (PICS) for both groups. In efforts to decrease symptoms of anxiety and depression, ICUs have implemented a variety of programs to reduce family distress.

**Methods:**

We conducted a systematic review of experimental studies which aimed to reduce stress related disorders in family members after the experience of having a patient admitted to the ICU. Multiple databases were searched for randomized controlled trials or nonrandomized comparative trials which targeted family members or surrogate decision makers. A total of 17 studies were identified for inclusion in the review representing 3471 participants.

**Results:**

We describe those interventions which we qualitatively assigned as “not passive,” or those which actively engaged the family to express themselves, as more likely to be successful in both the available pediatric and adult literature than interventions which we identified as “passive.” Studies which described active engagement of family members demonstrated comparative improvements in symptoms of depression, anxiety, and PTSD, as well as reduced hospital costs in the case of two studies.

**Discussion:**

This review may serve to aid in the development of future interventions targeted at reducing family stress and PICS following an ICU hospitalization.

## Introduction

An admission to the intensive care unit (ICU) is a stressful time for patients and their family members, but there is also increasing awareness about the lasting impact on emotional health after a patient’s hospitalization. In 2012, Davidson et al defined Post Intensive Care Syndrome-Family (PICS-F), reflecting the widespread prevalence of psychological distress and the presence of stress-related disorders up to four years after hospitalization^1^. As the psychological impact of hospitalization, particularly in an ICU, becomes an area of increasing research, many ICUs are implementing measures to combat PICS-F while the patient is still admitted to the ICU, such as family journals, individualized nursing support, and tailored educational materials [1,2].

The Society of Critical Care Medicine (SCCM) and the American Thoracic Society (ATS) have favored the implementation of support measures for families of ICU patients [3,4]. However, the impact of implementations of such programs on family members’ wellbeing, affect, and perceived quality of decision making has been poorly understood. We perform a systematic review of published literature to analyze the available data on the effects of the implementation of family support systems on quality of life, affect, emotional stress and quality of decision-making outcomes.

## Methods

This review was conducted according to PRISMA guidelines for reporting in systematic review.

### Data Sources

We searched the electronic databases of PubMED, EM-BASE, and CINAHL to identify empirical studies comparing outcomes without and with the implementation of family support measures for patients admitted in the ICU (last search on September 8, 2020). We searched using free text and MeSH terms including: critical care, decision-making (appendix 1). We identified 3428 possible publications. These citations were managed and screened using the Abstrackr webtool [5]. After a pilot round of 200 citations to ensure uniform application of screening criteria, all citations were screened independently by two reviewers. Conflicts were resolved in group discussions. All citations deemed eligible at the abstract level were screened in full text in duplicate and independently (SR and TA), and disagreements were resolved by a third reviewer (TT).

### Study Selection

Eligible studies enrolled adult family members of neonatal, pediatric, and adult patients hospitalized in a medical, neurological or surgical ICU for reasons other than traumatic brain injury or severe anoxic brain injury. These conditions were excluded because they reflect a minority of patients in an ICU census and are likely to have different emotional responses to the ICU process due to the traumatic or sudden nature of their admissions.

Eligible interventions targeted family members or surrogate decision makers for ICU patients and aimed to improve outcomes of knowledge, accuracy of risk perception, anxiety, stress or depression, or satisfaction with care and decision-making. We considered both formal decision aids and non-decision-aid supporting interventions which fulfilled the above criteria. We included randomized controlled trials (RCTs), and nonrandomized comparative studies with concurrent or historic controls. We excluded studies that did not report any information about the content (e.g., journal keeping, counseling) and delivery (timing, delivery format) of the intervention, or studies that did not report empirical outcomes.

### Data Extraction and Risk of Bias Assessment

Each study was extracted by an investigator, and confirmed by another, using pre-defined data extraction forms (Appendix 2). We used digitizing software to extract information from graphs (WebPlotDigitizer) [6]. We extracted information on the provenance of each paper (first author, title, journal and year of publication), its design (sample size, study type), characteristics of the patients, caregivers, the interventions, and data on outcomes, as described below. Three reviewers assessed the risk of bias of each study using the approach described in ROBINS I tool [7]. Any disagreements were resolved by discussion among all team members.

### Description of Data Extraction

We extracted results for validated instruments that measure *emotion and affect,* including four instruments which measured stress, two which measured anxiety, two which measured depression, and two which measured behavioral changes in children as indicators of stress (Table 1) [8, 9, 10, 11, 12, 13, 14, 15, 16, 17, 18, 19, 20]. Table 1 provides brief descriptions for these outcome scales and their validation.

For all scales, we recoded results so that increasing values imply worsening outcomes, but left each outcome reported over its typical range without recoding them on a common (e.g., 0-100) range. To help contextualize the magnitude of observed differences in each scale, we report empirically derived confidence intervals. If such estimates are not readily available, we report the interquartile range of scores.

**Table 1:** Instruments for measurement of stress and emotional affect

Tool Name	Intended Assessment	Scale Range	Scale Interpretation	Population	#Questions	Style	Comments
Parental Stressor Scale (PSS)	parent stress, negative feelings about children/ parenting	18-90	higher score indicates greater stress	Parents	18	self-reported	

Parenting Stress Index (PSI)	parent stress, positive and negative feelings about children/ parenting	0-100	higher score indicates greater stress, conflict	Parents	101	self-reported	

Neonatal Index of Parental Satisfaction (NIPS)	infant behavior	0-7	higher score indicates higher levels of satisfaction	Parents	30	self-reported	

Patient-Health Questionnaire (PHQ-9)	Depression	0-27	higher score indicates depression: Stratified according to severity- 0-4 Minimal or none Monitor; may not require treatment 5-9 Mild Use clinical judgment (symptom duration, functional impairment) to determine necessity of treatment 10-14 Moderate 15-19 Moderately severe Warrants active treatment with psychotherapy, medications, or combination 20-27 Severe	Adults	9	self-reported	

Generalized Anxiety Disorder Scale (GAD-7)	Generalized Anxiety Disorder	0-21	higher score indicates greater likelihood of clinically significant anxiety disorder Score Symptom Severity 5-9 Mild Monitor 10*-14 Moderate Possible clinically significant condition >15 Severe Active treatment probably warranted	Adults	7	self-reported	

Posttraumatic Stress Disorder Checklist (PCL)	PTSD	0-80	higher score indicates higher likelihood of PTSD provisional PTSD diagnosis can be made by treating each item rated as 2 = "Moderately" or higher as a symptom endorsed, then following the DSM-5 diagnostic rule which requires at least: 1 B item (questions 1-5), 1 C item (questions 6-7), 2 D items (questions 8-14), 2 E items (questions 15-20). Preliminary validation work is sufficient to make initial cut-point score suggestions, but this information may be subject to change. A PCL-5 cut-point score of 33 appears to be a reasonable value	Adults	20	self-reported	for civilians and military (not relevant for our study)

Hospital Anxiety and Depression Scales (HADS)	Anxiety and Depression	0-21 (for each scale-full HADS provides two separate scales)	higher score indicates higher likelihood of generalized anxiety or depression	Adults	14	self-reported	

State-Trait Anxiety Inventory (STAI)	Anxiety and Depression	20-80 (for each State and Trait)	higher score indicates higher likelihood of anxiety and stress	Adults, Care-givers	40	self-reported	20 trait-focused and 20 state-focused questions. Requires 6th grade reading level

Nurse Parent Support Tool (NPST)	parent perceptions of support, stress	1-5 per item	higher score indicates higher perceived support	Parents	21	self-reported	

Impact of Event Scale (IES)	subjective distress caused by traumatic events	0-88	higher score indicates higher likelihood of PTSD	Adults	22	self-reported	

### Data Synthesis and Analysis

We did not perform quantitative analyses (including sensitivity analyses) because of substantial diversity in populations, interventions, and outcomes. Instead, we characterized each intervention over key dimensions which we used as thematic entities to structure our descriptions and organize our conclusions. In all analyses we divided eligible studies into pediatric or adult patient populations.

### Categorization of Data

**Fig 1:**
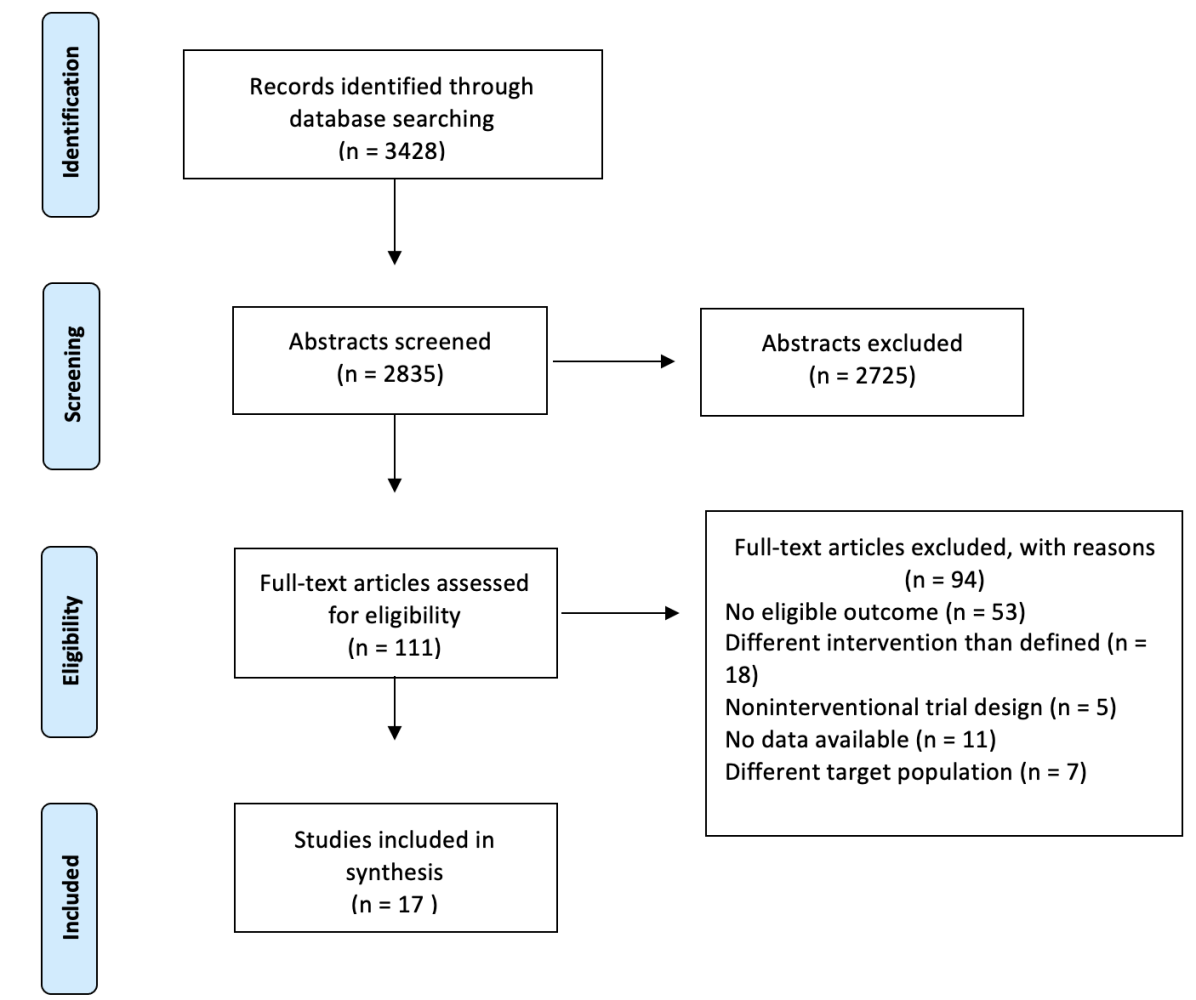
Study selection process, based on Preferred Reporting Items for Systematic Reviews and Meta-Analysis (PRISMA) guidelines.

Data was first examined and separated by the nature of the Intensive Care Unit in which the studies were conducted. Studies which were conducted in a Pediatric or Neonatal ICU were separated from studies conducted in an adult patient population, due to the different relationships of caregivers and assessment tools. In total, 17 studies were included in the final analysis (Figure 1).

### Categorization of Interventions

Interventions were initially described based on their content as well as the method of intervention delivery (Appendix 2). In assessing content, we categorized interventions as containing information regarding resources/procedures, containing general information pertaining to ICU care, containing information tailored to the patient, encouraging family participation in care, formal counseling, peer counseling, or use of navigator/facilitator support. The content of each intervention was briefly described for each relevant group. Intervention delivery was described in terms of personnel and timing with regards to the patient’s hospitalization. When available, the training of personnel who delivered the intervention was included, as well as their role on the ICU team, if one existed.

Further, we characterized each the intervention as “passive” or “not passive”. Passive interventions were those that focused on delivery of information, but without active engagement of the family or patient proxies in some way. Non-passive interventions allowed or encouraged engagement of the family, e.g., through participation in care, narrative writing.

## Results

### Study inclusion

Our literature search yielded 3428 publications for possible inclusion (Figure 1). Of these, 2835 were considered relevant for full review after abstract review by two of the authors (TA and SR). For any abstracts where there was uncertainty regarding inclusion, a third author (TT) reviewed the abstract and all three authors agreed on inclusion or exclusion. Abstract review identified 110 articles which were subsequently assessed in full. Of these, 17 were identified and included in the analysis. Of the 17, 8 pertained to pediatric populations (6 in the neonatal ICU and 2 in the pediatric ICU) and 9 to the adult ICU populations.

### Study Description and Analysis

The 17 studies that were eligible for inclusion were systematically reviewed and summarized in tabular format for the study design and characteristics, intervention content, delivery and timing, and main findings. These were reviewed by three of the authors (TA, SR, and TT) for fidelity and the results are summarized in Table 2 for pediatric and Table 3 for adult populations. Of the 17 studies included, 13 of them were randomized control trials, and 4 were defined as quasi-experimental, totaling 3471 research participants. One of the studies (Curley) was published in 1988, 5 trails were published between 2004 and 2008 (Melnyk, Browne, Moreau, Kloos, and Lautrette), and the remaining trials were published after 2011 [21, 23, 24, 31, 32, 33]. Nine of the studies included were completed in the United States (Kloos, Carson, Curtis, White, Clarke, Curley, Brown, Melnyk, Amass), 5 in Europe (Weis, Garrouste-Orgeas, Franck, Moreau, Lautrette), 1 in Iran (Kadivar), 1 in Australia (Abdel-Latif), and 1 in China (Chiang)[22, 23, 24, 25, 26, 27, 28, 29, 30, 31, 32, 33, 34, 35, 36, 37]. For one study (Amass), the relevant data was obtained from the author and not included in the original publication [33].

**Table 2:** Included Studies with outcome measures by intervention type, Not Passive or Passive

Setting/Year	Author		Emotional Outcome Tool mean result, control (95% Cl) versus experimental (95% Cl)				Satisfication Outcomes Mean Results, control (95% Cl) versus experimental (95% Cl)
**Pediatric - Not Passive**		**PSS:NICU**	**PSS**	**NIPS**	**STAI**	**PSI-P**	**NPST**

2005	Browne J, Talmi A						

2017	Kadivar M, et al			102.2 (94- 110) VS 137 (132- 142)			

1988	Curley M		2.66 vs 1.92 (Cl unable to calculate)				

2004	Melnyk B, et al				39.1 (36-42.2) vs 36(33.7- 38.3)	7.4(6.12-8.68) vs 5.6(4.76-6.44)	

**Pediatric - Passive**							

2011	Franck L, et al	2.57 (2.44- 2.7) VS 2.58 (2.44- 2.72)					

2015	Abdel-Latif M, et al		3.47 (3.3- 3.64) VS 3.47(3.26-3.61)				

2015	Clarke-Pounder J, et al				Anxiety change -4.2 (-6.89-1.51) vs 0.33 (-5.67-6.34)		

2013	Weiss J, et al	2.84(2.61-3.07) VS 2.7 (2.52-4.47)					4.16 (3.97-4.35) vs 4.14 (4.01-4.27)

**Table 3:** Included Adult Studies with outcome measures by intervention type, Not Passive or Passive

Setting/ Year	Author		Emotional Outcome Tool mean result, control (95% CI) versus experimental (95% CI)					Satisfaction Outcomes Mean Results, control (SD) versus experimental (SD)	Resource Utilization Mean Results, control (SD) vs experimental (SD)		
**Adult- Not Passive**		**STAI**	**HADS**	**PHQ-9**	**GAD-7**	**C-DASS**	**IES-r PCL PPPC**	**FSICU-24**	**LOS: ICU**	**LOS: Hospital**	**Cost of care (thousands)**

2018	White D, et al		12.1 (11.54- 12.66) vs 11.7 (11.04­12.36)				20.7 (19.53 - 21.87) vs 1.8 (0.7) vs 20.5 (18.981.6 (0.6) 22.02)		8.8 , (8.229.38) vs 8.1 (7.38- 8.82) ' '	15.5 (14.23 - 16.77) vs 11.8 (10.7­12.9)	

2016	Curtis JR, et al			4.7 vs 2.4 (-0.431-0.43)	2.7 vs 1.8 (-3.11.32)		30.6 vs 27.1 (-7.12- 0.10)		21.4 vs 17.4 (-0.14- 0.46)	32.6 vs 24.1 (0.24- 0.86)	ICU: 75.85 vs 51.1 (48.68--0.9) Hospital: 170.7 vs 123.1 (-90.48--4.67)

2007	Lautrette A, et al		17 (IQR 11-25) vs 11 (IQR 8-18)				39 (IQR 25-48) vs 27 (IQR 18-42)				

2008	Kloos J and Daly B	39.53 (36.23 - 42.83) vs 40.42 (37.83 - 43.01)									

2020	Amass T et al		12.31 (10.94- 13.67) vs 13.16 (12.214.13)				27.2 (23.56- 30.84) vs 12.3 (10.94- 13.67)	85.44 (87.31- 91.95) vs 89.63 (83.14- 87.74)			

**Adult - Passive**											

2004	Moreau D, et al		Total 21 (ND) vs 22 (ND) Anxiety 13 (ND) vs 9 (ND) Depression 8 (ND) vs 9 (ND)								

2016	Carson S, et al		Anxiety: 6.4 (5.72 - 7.08) vs 7.2 (6.54 - 7.84) Depression: 5 (4.34- 5.66) vs 4.9 (4.29- 5.51)				25.6 (23 - 28.2) vs 20.7 (18.03-23.37)	81.1 (78.3- 83.9) vs 84.3 (81.387.3)			

2017	Chiang V, et al					Anxiety: 7.71 (4.92- 10.5) vs 7.23 (4.9-9.56) Depression: 7.94 (4.84- 11.04) vs 6.1 (3.88-8.32)					

2016	Garrouste- Orgeas M, et al		Anxiety: 8 (IQR 4.5 - 12) vs 4 (IQR 1-9) Depression: 5.5 (IQR 1- 11.5) vs 2 (IQR 0- 6)				24 (ND) vs 21 (ND)				

### Pediatric Results

Eight studies evaluated participants associated with pediatric patient populations. Outcomes measured in these populations included several validated scales. The Parental Stress Score (PSS) was used in 5 of the studies, which results with scores between 18 and 90, with higher scores indicating more stress [8]. The Parenting Stress Index (PSI) was used by Browne et al and Melnyk et al with higher scores indicating more parental stress [9]. One study (Kadivar) used the Neonatal Index of Parent Satisfaction (NIPS) which results scores between 24 and 168 with higher scores indicating more parental satisfaction [10]. Two studies (Clarke-Pounder and Melnyk) used the State-Trait Anxiety Index (STAI), which is scored between 20-80, with higher scores indicating higher state or trait anxiety levels [11]. Finally, Weis et al used the Nurse Parent Support Tool (NPST) in addition to the PSS-NICU, which generates scores between 21-105, with higher scores indicating more positive experiences of support [12]. Of note, Melnyk et al had several time points of measure ranging from during the intervention to 12 months post-intervention. To allow for comparison to the other studies included, the 6-month observations were included in the results of this review.

### Not Passive

There were 4 pediatric studies that were determined to have an intervention that was not passive. Browne found that education of mothers with infants hospitalized in the NICU regarding their infants improved overall stress scales [21]. The study authors were contacted to request original PSI data for this analysis but were unable to provide this information. Without the original data, the study was subsequently excluded from further analysis. Kadivar demonstrated that with narrative writing, parents’ satisfaction was higher by day 10 in the intervention group vs the control as measured by NIPS [22]. Curley et al showed that a model that had nurses engage family members to gain understanding of family and patient wishes reduced parental stress as measured by the PSS:PICU [23]. Melynk et al used an intervention titled “Creating Opportunities for Parent Empowerment” (COPE), which increased parental knowledge and participation in childcare and found trends towards improvement across several domains, though none were significant [24]. At 6 months they showed a reduction in parental stress in the intervention group, as well as lower anxiety.

### Passive

Four pediatric studies were categorized as passive. Franck et al found no change in parental stress at one week as measured by PSS:NICU with delivery of a booklet that contained detailed information about pain and comforting along with demonstrations by a research nurse [25]. A second study (Abdel-Latif et al) included parents on rounds and found no difference in parental stress with the PSS or subscales [26]. Clarke-Pounder et al employed an intervention which gathered information from parents about care preferences and placed them in the chart for the provider [27]. This intervention demonstrated more reduction in anxiety between baseline and 2 weeks in the control group, but no change in the intervention group. Finally, Weis et al offered reflection sheets to the parents and showed no change in parental stress or satisfaction through this intervention [28].

### Adult Results

There were 9 studies that focused on family members of adult patients. Outcomes were also evaluated with several different validated tools. Daly et al utilized the STAI, described in the pediatric results section [11]. Six of the studies (White, Garrouste-Orgeas, Lautrette, Moreau, Carson, and Amass) used the Hospital Anxiety and Depression Scale (HADS) which results scores between 0 and 21 for anxiety or depression, with higher scores indicating more symptoms consistent with either diagnosis [13]. Five studies (White, Garrouste-Orgeas, Lautrette, Carson and Amass) also used the Impact of Events Scale- revised (IES-r), a 22-item scale producing scores between 0 and 88, with higher scores being more consistent with symptoms of Post-Traumatic Stress Disorder (PTSD) [14]. In addition to the above two scales, White et al utilized the Patient Perception of Patient Centeredness (PPPC, modified for surrogates) which produces scores from 1 to 4 with lower scores indicating more patient/family centered care [15]. Carson et al and Amass et al also evaluated family satisfaction using the Family Satisfaction in the ICU-24 (FS-ICU24) questionnaire, scored between 24 and 100 with higher scores indicating more satisfaction [16]. Chiang et al utilized the Depression Anxiety Stress Scale- Chinese (C-DASS), a 21-item scale scored between 0-21 for each depression, anxiety, and stress, with higher scores indicating higher levels of each diagnosis [17]. The final paper to utilize multiple scales, Curtis et al, used the Patient Health Questionnaire (PHQ-9), the Generalized Anxiety Disorder-7 (GAD-7) survey, and the PTSD Checklist Civilian Version (PCL) to evaluate for symptoms of depression, anxiety, and PTSD, respectively [18, 19, 20]. For each metric, a higher score indicates more symptoms of the measured state. Curtis et al and White et al additionally evaluated length of stay (LOS) and cost of care.

### Adult Not Passive

Five of the included adult studies were categorized as instituting interventions that were not passive. White et al found that with engaging the family members with a “PARTNER” nurse, a nurse specially trained on supporting the family and meeting with them daily as well as coordinating provider team meetings, there were no changes in symptoms of anxiety or depression or PTSD compared to the control [29]. The intervention did demonstrate a significant reduction in ICU LOS and hospital LOS as compared to the control group. Using a “nurse navigator,” a nurse specially trained in mediation and communication techniques who met with the family to engage them and help express their concerns during provider meetings, Curtis et al demonstrated a significant reduction in symptoms of depression in the intervention vs the control 6 months after discharge of the patient, without a significant change in symptoms of anxiety or PTSD over the same time period [30]. The intervention did demonstrate a significant reduction in hospital LOS without a significant change in ICU LOS, and additionally demonstrated a significant reduction in both ICU and hospital costs. Lautrette et al implemented a physician training on the VALUE mnemonic (supplemental index), which trained physicians to allow family members to express themselves more during the family meetings as well as elicit and validate their feelings [31]. With this intervention, the authors reported a significant reduction in anxiety and depression and PTSD in the intervention vs control 90 days after discharge of the patient from the ICU. Kloos and Daly reported that with an intervention which instructed family members to complete a journal of both events (eg surgery timing, patient condition) and feelings, there was no change in anxiety state in the intervention as compared to the control [32]. Finally, Amass et al demonstrated that active engagement of family members in patient care resulted in a significant reduction in symptoms of PTSD in the intervention compared to the control group 90 days after patient discharge [33]. Family members who participated in the intervention seemed to have higher overall satisfaction with the care that their loved one received in the ICU than individuals who did not participate in the intervention, although this did not reach the level of statistical significance. However, the study noted no significant change in depression or anxiety scores in the intervention vs the control at 90 days post discharge of the patients.

### Adult Passive

There were 4 studies of adult populations categorized as passive. Moreau et al reported no difference in anxiety or depression scores as measured by HADS when information was delivered by a senior physician as compared to a junior physician [34]. When compared with usual care, Carson et al reported that palliative care lead meetings in the ICU demonstrated an increase in symptoms of PTSD but did not alter symptoms of depression or anxiety [35]. They additionally reported no difference in satisfaction scores using the FSICU-24. Chiang et al reported a reduction in depression symptoms and no difference in symptoms of anxiety when a nurse delivered a tablet to the family loaded with patient disease-specific information [36]. Finally, Garrouste-Orgeas et al report a reduction in anxiety and depression scores on the HADS when nurses were trained to deliver information to the families and participation with the family meetings [37]. They also report a reduction in PTSD symptoms as measured by IES-r scores.

## Discussion

While analyzing the support systems instituted by these diverse intensive care units, data consistently demonstrated two general approaches to family support. In one category, measures were instituted by the units themselves to mitigate distress, and family participation was primarily in the passive form. In the other, families were encouraged to actively engage in the patient’s care and understand the nuances of hospitalization. When the interventions instituted are divided into these 2 general categories, it appears that those which allow a family member to express themselves are more likely to be successful.

Considering both the pediatric and adult literature, there are 9 studies which report positive results. Of the four pediatric studies, two provided direct opportunities for parental expression (Kadivar and Melnyk) in either the form of narrative writing or a program (COPE) targeted at parental involvement [22, 24]. The remaining two (Browne and Curley) with improvement in outcomes offered patient specific information and teaching targeted at allowing the parent to engage with their child in a family-specific way, as opposed to a more formulaic mechanism [21,23]. The pediatric literature that either showed no difference in outcomes or worsening in outcomes presented interventions that did not clearly allow individual expression. Franck et al offered generic information about infant pain and comforting techniques while Abdel-Latif et al allowed presence on rounds [25, 26]. Weis et al appear to have offered “reflection sheets” for self-expression but were coupled with structured discussions and did not show a reduction in parental stress [28]. However, an important consideration in this study is that the same nurses delivering the intervention were caring for patients in both the intervention and control groups which creates concern for confounding. The final pediatric study which demonstrated worsening of parental satisfaction with the intervention (Clarke-Pounder) attempted to employ tailored communication based on documented communication preferences [27]. Notably, it discusses the lack of attention to emotional or psychosocial aspects during communications, suggesting that a focus on the delivery of information solely as it pertains to the medical condition of the child is not beneficial to the parents.

Amongst the studies included from adult populations, six reported positive findings, and five of these utilized interventions which offered clear opportunities for family expression. Both Curtis et al and White et al engaged a nurse facilitator/communicator and demonstrated either a reduction in stress related symptoms (Curtis), or an increase in satisfaction (White), with both demonstrating significant reductions in hospital length of stay and hospital cost [29,30]. Lautrette et al trained physicians on a specific mnemonic which encouraged more active listening and eliciting family expression, which subsequently resulted in reduced PTSD, depression and anxiety scores for family members [31]. Garrouste-Orgeas et al allowed for more family-directed expression including the bedside nurses in family meetings, as opposed to meetings in which the sole care team representative was a physician [37]. The presence of a bedside nurse in these meetings appeared to allow the nurse to serve as an advocate for the family to express their wishes and those of the patient and led to reduced family depression and anxiety scores. Amass et al reported that encouraging bedside participation of the family in the care of the ICU patient allowed for family members to express themselves and care for the patient in a family-centered way, and reported reduced symptoms of PTSD at follow up [33]. In the final positive study, Chiang et al used a tablet for communicating information and demonstrated a reduction in depression in the family members [36].

As in the pediatric literature, the two studies without positive results either did not allow for family member self-expression (Moreau), but rather dictated which physician delivered information (junior vs senior physician), or did offer self-expression (Kloos, utilizing diaries) but measured the outcome of interest at only 3 days [32,34]. This contrasts sharply with the majority of other studies which chose to additionally measure outcomes after at least 1 month, if not 3 or 6 months. In the adult study with negative results, Carson et al found that palliative care led family meetings in the ICU lead to higher rates of PTSD than usual care [35]. This aligns with the framework that an intervention targeting changes in communication towards the patient and family, even by experts in the field, does not improve stress symptoms, and may in fact worsen them.

While this review serves to summarize available research on supporting family members of patients hospitalized in the ICU, it also serves to identify a lack of clear information and direction. A 2020 systematic review of PICS-Family in a strictly adult ICU population identified interventions during and after an ICU hospitalization similarly identified a benefit of proactive communication measures, while additionally noting the potential for some interventions to worsen PICS [38]. Our review provides a novel framework that categorizes interventions by the level of active engagement of family members. Through categorization of interventions in this way, we have been able to identify a pattern of ICU engagement which may prove of greater benefit to families and serve as an area of focus for future research. In addition, this review includes pediatric patients and strengthens the theory that non-passive interventions lead to the greatest reduction in family stress across patient populations, rather than solely focusing on adult patients. By expanding the population included in this review, we hope to bridge a gap between adult and pediatric literature, to allow for more effective family engagement across ICUs, regardless of the population served.

For practicing providers, analysis of positive studies may identify areas for focus while further research is being conducted. We recommend that providers focus on active engagement of the patient families, and on eliciting additional information about the patient for whom they are caring. When possible, the presence of bedside nurses at family meetings may offer support to families who see them as important patient advocates. While the positive studies demonstrate the importance of family engagement, the presence of visitation restrictions during the COVID-19 pandemic has resulted in an added challenge to family involvement. Providers may be able to arrange video conferences when not possible for family members to be at bedside. In addition, units may need to consider the adjustment of visitation policies in order to allow for family members to more effectively engage in the care of critically ill patients when able.

It would seem that interventions aimed at empowering family members to express themselves may be of larger benefit; however, the strength of these studies is moderate, and variable design, particularly with regards to assessment tools and time to follow-up, makes comprehensive analysis challenging. In addition, to relative paucity of literature focused on this issue to date makes a clear argument for future studies dedicated at understanding and reducing PICS-F. The visitation restrictions placed by many hospitals due to the COVID-19 pandemic have additionally highlighted the need for support tools for families. Based on this review, future studies may consider interventions such as those described above in order to help patients and their families cope with the emotional ramifications of an ICU hospitalization.
